# Impact of KRAS mutation status on the efficacy of immunotherapy in lung cancer brain metastases

**DOI:** 10.1038/s41598-021-97566-z

**Published:** 2021-09-13

**Authors:** Adam Lauko, Rupesh Kotecha, Addison Barnett, Hong Li, Vineeth Tatineni, Assad Ali, Pradnya Patil, Alireza M. Mohammadi, Samuel T. Chao, Erin S. Murphy, Lilyana Angelov, John H. Suh, Gene H. Barnett, Nathan A. Pennell, Manmeet S. Ahluwalia

**Affiliations:** 1 Case Western Reserve University School of Medicine MSTP, Cleveland, OH USA; 2 Department of Radiation Oncology, Miami Cancer Institute, Baptist Health South Florida, Miami, FL USA; 3 Herbert Wertheim College of Medicine, Florida International University, Miami, FL USA; 4 Rosa Ella Burkhart Brain Tumor and Neuro-Oncology Center, Taussig Cancer Institute, Cleveland, OH USA; 5 Department of Quantitative Health Sciences, Lerner Research Institute, Cleveland Clinic, Cleveland, OH USA; 6 Department of Internal Medicine, Summa Health, Akron City Hospital, Akron, OH USA; 7 Department of Medical Oncology, Taussig Cancer Institute, Cleveland Clinic, Cleveland, OH USA; 8 Department of Neurological Surgery, Neurological Institute, Cleveland Clinic, Cleveland, OH USA; 9 Department of Radiation Oncology, Taussig Cancer Institute, Cleveland Clinic, Cleveland, OH USA; 10 Department of Medical Oncology, Miami Cancer Institute, Baptist Health South Florida, 8900 North Kendall Drive, Miami, FL 33176 USA

**Keywords:** CNS cancer, Non-small-cell lung cancer

## Abstract

Immune checkpoint inhibitors (ICIs) have resulted in improved outcomes in non-small cell lung cancer (NSCLC) patients. However, data demonstrating the efficacy of ICIs in NSCLC brain metastases (NSCLCBM) is limited. We analyzed overall survival (OS) in patients with NSCLCBM treated with ICIs within 90 days of NSCLCBM diagnosis (ICI-90) and compared them to patients who never received ICIs (no-ICI). We reviewed 800 patients with LCBM who were diagnosed between 2010 and 2019 at a major tertiary care institution, 97% of whom received stereotactic radiosurgery (SRS) for local treatment of BM. OS from BM was compared between the ICI-90 and no-ICI groups using the Log-Rank test and Cox proportional-hazards model. Additionally, the impact of *KRAS* mutational status on the efficacy of ICI was investigated. After accounting for known prognostic factors, ICI-90 in addition to SRS led to significantly improved OS compared to no-ICI (12.5 months vs 9.1, *p* < 0.001). In the 109 patients who had both a known PD-L1 expression and *KRAS* status, 80.4% of patients with *KRAS* mutation had PD-L1 expression vs 61.9% in wild-type *KRAS* patients (*p* = 0.04). In patients without a *KRAS* mutation, there was no difference in OS between the ICI-90 vs no-ICI cohort with a one-year survival of 60.2% vs 54.8% (*p* = 0.84). However, in patients with a *KRAS* mutation, ICI-90 led to a one-year survival of 60.4% vs 34.1% (*p* = 0.004). Patients with NSCLCBM who received ICI-90 had improved OS compared to no-ICI patients. Additionally, this benefit appears to be observed primarily in patients with *KRAS* mutations that may drive the overall benefit, which should be taken into account in the development of future trials.

## Introduction

Lung cancer is the most common primary tumor that metastasizes to the brain, accounting for approximately 50% of brain metastases (BM)^1^. For patients without actionable mutations, systemic chemotherapy has shown little intracranial efficacy, and traditionally, non-small cell lung cancer (NSCLC) BM have been managed with local treatments, such as resection and radiotherapy.

Exciting developments in cancer therapeutics targeting the PD-1 or PD-L1 axis have led to the approval of multiple immune checkpoint inhibitors (ICIs) as standard of care options in the first line setting^2^ and as salvage options after failure of first-line chemotherapy^3^. However, given the historically poor prognosis of patients with NSCLCBM, these patients were excluded from many of the initial trials unless they had previously treated and stable intracranial disease^2–4^. Recently, several retrospective series and a single center phase 2 clinical trial have demonstrated limited intracranial efficacy with ICIs alone, ranging from 12 to 40%^5–7^. Additionally, there is newer evidence that a combination approach with stereotactic radiosurgery (SRS) and ICI may be more beneficial for intracranial control, as this has been shown to have better intracranial response over either therapy alone^8^. Additionally, many researchers are interested in possible increased rates of the abscopal effect when fractional dose radiation and ICIs are given concordantly^9^.

*KRAS* mutations are present in approximately 30% of NSCLC adenocarcinomas and have been associated with a poorer prognosis when compared to wild-type tumors^10–12^. Until May 2021, there was no FDA approved targeted therapies towards *KRAS* mutations for patients with NSCLC. Patients frequently receive ICIs as standard-of-care treatments based on PD-L1 expression, regardless of *KRAS* mutation status. There is also evidence that *KRAS* mutations lead to increased expression of PD-L1, a known predictor of response to ICIs^13,14^; however, impact of *KRAS* mutation status on treatment outcome for patients with NSCLCBM has yet to be empirically demonstrated.

In this study, we analyzed a large population of patients with NSCLCBM who were treated at a single tertiary center. We compared the survival of those who received ICI after the diagnosis of BM to those who did not receive ICI within this timeframe. Additionally, the impact of *KRAS* mutation status on the efficacy of ICI was investigated. We hypothesized that patients treated with ICIs after the diagnosis of BM would have an improved survival and that within the ICI group, those with *KRAS* mutations would have improved survival over those with *KRAS* wild-type disease.

## Methods

### Patient selection and treatment

This retrospective cohort study included patients treated for NSCLCBM at a single tertiary care center from 2010 to 2019. This study was approved by the Cleveland Clinic Foundation institutional review board and received an informed consent waiver due to the retrospective nature of the study. All methods were carried out in accordance with relevant guidelines and regulations. Patient characteristics, diagnostic information, and treatment details were abstracted from the shared electronic medical record.

All the patients’ treatments were extracted after diagnosis of the primary cancer. This included SRS, whole-brain radiation therapy (WBRT), surgery, as well as any systemic therapies, including targeted therapies, chemotherapy, and ICI. Patients’ PD-L1 expression were extracted from pathology reports of tissue, primarily from the lung. Patients who had documented *EGFR* or *ALK* mutations who received a targeted therapy towards these mutations were excluded from this analysis. Patients who underwent resection of their intracranial tumors or diagnosed with small cell lung cancer were also excluded. All treatment decisions were made by the multidisciplinary team and were based on current national guideline standards at the time. Dosing schedule for Nivolumab was 3 mg/kg or 240 mg flat dose every other week and 2 mg/kg or 200 mg flat does every three weeks or 400 mg flat dose every six weeks for pembrolizumab.

Patients were divided into three groups based on if and when they were treated with ICIs. Those treated with ICIs within 90 days of the diagnosis of BM were classified as ICI-90, and those treated with ICI greater than 90 days after diagnosis of BM were classified as G90. Patients who did not receive ICI after diagnosis of BM at any point were classified as no-ICI.

### Outcome measures

Overall survival (OS) was determined from the date of diagnosis of BM until the date of death. Patients who were still alive at the day of collection or lost to follow-up were censored at the last follow-up date.

### Statistical analysis

All variables included in this analysis had < 5% missing except for Karnofsky Performance Status (KPS) at diagnosis (15% missing). Several numerical variables were also grouped as categorical and both their numerical and categorical forms were evaluated. This included age (< 65 and ≥ 65), number of SRS treatments (0–1 and ≥ 2) (only 3% of patients did not receive SRS), lesion number at diagnosis (1 and ≥ 2) and KPS (< 90 and ≥ 90). Patient demographic and clinical characteristics were compared using Chi-square test for categorical variables and Kruskal–Wallis test for numerical variables. If any differences between the three groups were detected, no-ICI and ICI-90 were then compared. Times from BM diagnosis to death or last contact were calculated for OS analysis. Patients who died after 2 years or followed more than 2 years were censored at 2 years.

Patient demographics and clinical characteristics that were potentially associated with OS (*p* < 0.05) among BM patients who did not have surgery were identified in univariate analysis using Kaplan–Meier method and simple Cox hazard models. Since identified factors potentially associated with OS were previously reported to associate with survival or commonly included in lung cancer BM studies^15^, they were included in the multivariate analysis of OS between ICI-90 and no ICI. A 1:1 propensity score (PS) matched analysis between no-ICI and ICI-90 was also performed to correct potential treatment selection biases. The matched pairs were identified from a logistic regression model with the Greedy method that included age, KPS, extracranial metastases (EC-mets), lesion number, and SRS number with exact match on EC-mets. Kaplan–Meier method was utilized to compare OS between matched ICI-90 and no-ICI group.

A secondary analysis explored ICI effect on OS by *KRAS* status. Among patients who had a known *KRAS* mutation status, Cox proportional hazard model with an ICI-*KRAS* interaction term was utilized to determine the impact of *KRAS* mutation status on ICI efficacy.

All analyses were performed using SAS version 9.4, two-sided p values are presented. A *p* < 0.05 was considered as statistically significant.

## Results

### Patient characteristics

**Figure 1:**
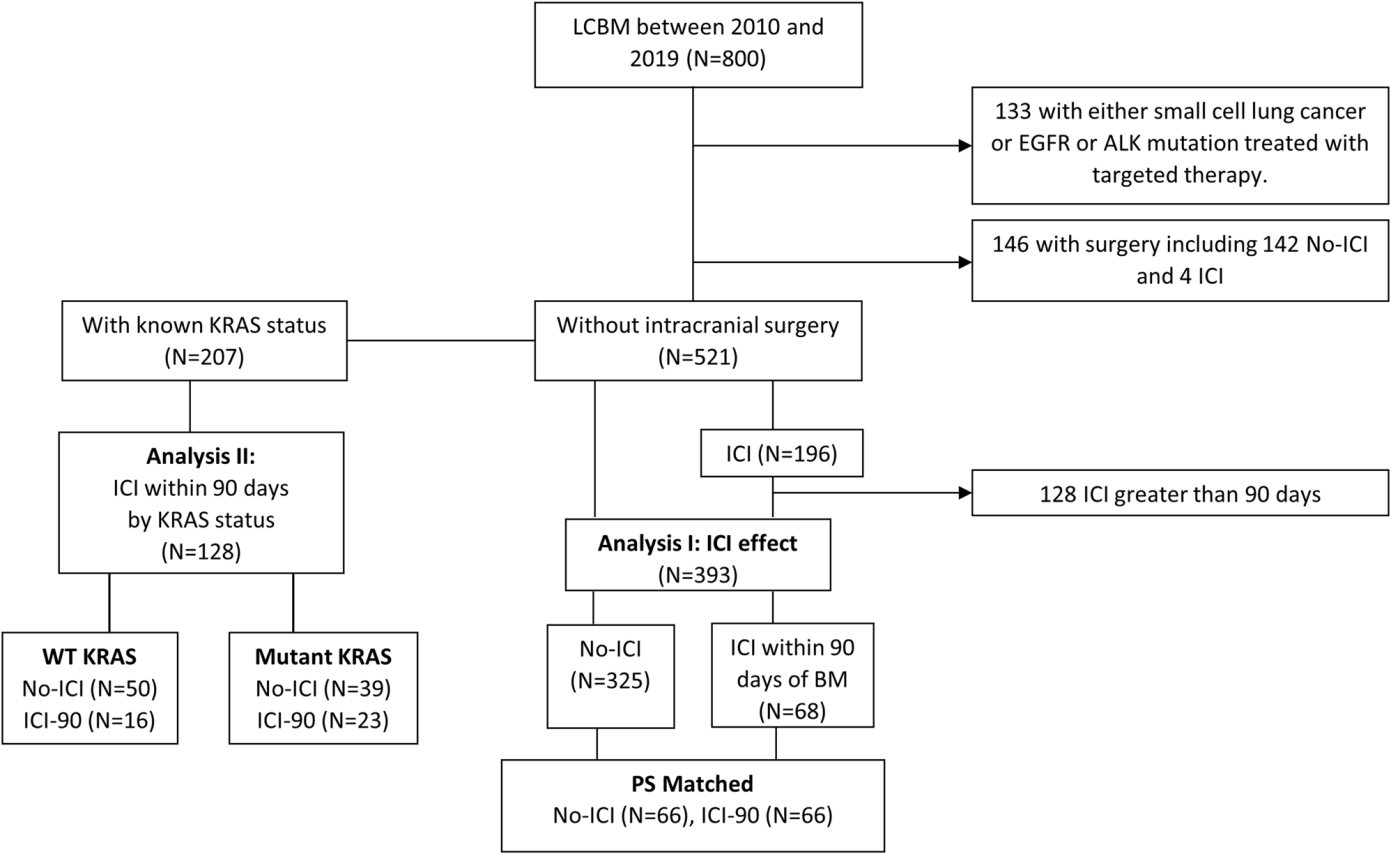
Flow chart of the study population. ICI = immune checkpoint inhibitor; BM = Brain Metastases; LCBM = lung cancer brain metastases.

A total of 800 patients were diagnosed with lung cancer BM at our tertiary center between 2010 and 2019. Ninety-seven percent of the patients in this cohort received SRS for intracranial treatment of BM. One hundred and thirty-three patients with small cell lung cancer or NSCLC that was treated with targeted therapy against an *EGFR* mutation or *ALK* translocations were excluded from the analysis, leaving 667 patients. Additionally, 146 patients underwent an intracranial tumor resection, with four of these patients receiving ICIs versus 142 never receiving ICIs. Because of this heavily skewed distribution, these patients were excluded as well. This left 521 patients with the general characteristics outlined in Table 1. Twenty-five percent (n = 128) of patients received ICI greater than 90 days after the diagnosis of BM, 13% (n = 68) received ICI within 90 days of diagnosis of BM, and 62% (n = 325) of patients never received ICI (Fig. 1).

**Table 1:** Patient characteristics at diagnosis of brain metastasis.

Factor	Total (N = 521)	No-ICI (N = 325)	ICI-90 (N = 68)	G90 (N = 128)	*p* value overall/No-ICI vs IC-I90
Age	62.7 [56.9, 70.6]	63.0 [57.1, 70.7]	60.4 [54.1, 70.3]	62.2 [57.1, 70.3]	0.29
Age, ≥ 65	214 (41.1)	139 (42.8)	22 (32.4)	53 (41.4)	0.28
Sex: male	262 (50.3)	174 (53.5)	32 (47.1)	56 (43.8)	0.15
Race: white	439 (84.3)	272 (83.7)	55 (80.9)	112 (87.5)	0.43
KPS	80.0 [80.0, 90.0]	80.0 [70.0, 90.0]	80.0 [70.0, 90.0]	90.0 [80.0, 90.0]	**< 0.001/0.86**
KPS ≥ 90					**< 0.001**
< 90	221 (42.4)	140 (43.1)	38 (55.9)	43 (33.6)	
≥ 90	220 (42.2)	112 (34.5)	29 (42.6)	79 (61.7)	
Unknown	80 (15.4)	73 (22.5)	1 (1.5)	6 (4.7)	
Lesion number	2.0 [1.00, 3.0]	1.00 [1.00, 3.0]	2.0 [1.00, 3.0]	2.0 [1.00, 3.0]	0.27
Lesion ≥ 2	261 (50.3)	153 (47.4)	37 (54.4)	71 (55.5)	0.23
Number of SRS	1.00 [1.00, 2.0]	1.00 [1.00, 2.0]	1.00 [1.00, 2.0]	2.0 [1.00, 2.0]	**< 0.001/0.64**
SRS ≥ 2	164 (32.2)	81 (25.7)	17 (25.4)	66 (52.0)	**< 0.001**
Histology: adenocarcinoma	371 (73.9)	216 (70.6)	52 (76.5)	103 (80.5)	0.089
EC-mets	238 (46.6)	139 (44.0)	37 (55.2)	62 (48.4)	0.22
WBRT	131 (25.1)	86 (26.5)	11 (16.2)	34 (26.6)	0.19
KRAS status					**< 0.001/< 0.001**
Negative	109 (20.9)	50 (15.4)	16 (23.5)	43 (33.6)	
Positive	98 (18.8)	39 (12.0)	23 (33.8)	36 (28.1)	
Missing	314 (60.3)	236 (72.6)	29 (42.6)	49 (38.3)	
PDL1 status					**< 0.001/< 0.001**
Negative	30 (5.8)	16 (4.9)	4 (5.9)	10 (7.8)	
Positive	93 (17.9)	27 (8.3)	40 (58.8)	26 (20.3)	
Missing	398 (76.4)	282 (86.8)	24 (35.3)	92 (71.9)	

Data not available for all subjects. Missing values: KPS = 80, lesion number = 2, srs number = 12, Histology = 19, Extracranial Metastases = 10. Statistics presented as Median [P25, P75] or N (column %). Significant differences (p < 0.05) are bolded.

Patient characteristics between those receiving ICI compared to those who never received ICI are outlined in Table 1. There was no statistical difference in age, sex, or race between the two groups (*p* > 0.05). The median KPS at diagnosis was 80 for both groups. The median number of lesions at the diagnosis of BM was 1 in no-ICI group and 2 in the ICI-90, however this variance was not statistically different (*p* = 0.27). Of the patients in the ICI-90 cohort, 55.2% had an EC-mets compared to only 44.0% in the no-ICI group.

Demographics and clinical factors that may associate with OS were first identified by univariate analysis across the 521 patients. KPS below 90 (*p* < 0.001), age older than 65 (*p* = 0.005), presence of EC-mets at diagnosis of BM (*p* = 0.02), adenocarcinoma histology (*p* < 0.001), and male sex (*p* = 0.02) were significant predictors of worse OS. However, the number of intracranial lesions at diagnosis of BM was not found to be statistically significant (*p* = 0.72) (Table 2).

**Table 2:** Univariate﻿ analysis of demographics and clinical characteristics.

Variable	N	Events	Median months	1-Year survival % (95% CI)	log-rank *p* value	Cox univariate hazard ratio (95% CI)	Cox univariate Wald p-value
**Sex**					**0.023**		
Female	**259**	**157 (61%)**	**13.4**	**58.4 (52.3, 64.5)**		**–**	
Male	**262**	**178 (68%)**	**11.1**	**48.6 (42.4, 54.8)**		**1.28 (1.03, 1.59)**	**0.024**
**Race**					0.75		
Non-white	82	51 (62%)	12.3	52.0 (40.9, 63.1)		–	
White	439	284 (65%)	12.7	53.8 (49.0, 58.5)		0.95 (0.71, 1.28)	0.75
**Age**					**0.005**		
< 65	**307**	**186 (61%)**	**13.8**	**56.8 (51.1, 62.4)**		**–**	
≥ 65	**214**	**149 (70%)**	**10.5**	**48.8 (42.0, 55.6)**		**1.36 (1.10, 1.69)**	**0.005**
**KPS**					**< 0.001**		
< 90	**221**	**157 (71%)**	**8.9**	**40.8 (34.2, 47.4)**		**1.97 (1.55, 2.50)**	**< 0.001**
≥ 90	**220**	**119 (54%)**	**18.4**	**66.8 (60.5, 73.1)**		**–**	
Missing	**80**	**59 (74%)**	**12.1**	**51.3 (40.0, 62.6)**		**1.65 (1.21, 2.26)**	**0.002**
**Lesion number**					0.49		
1	258	160 (62%)	13.3	55.7 (49.4, 61.9)		–	
≥ 2	261	173 (66%)	12.3	51.4 (45.2, 57.6)		1.08 (0.87, 1.34)	0.49
**SRS**					**< 0.001**		
0–1	**345**	**245 (71%)**	**9.8**	**43.4 (38.1, 48.8)**		**–**	
≥ 2	**164**	**82 (50%)**	**21.7**	**75.1 (68.4, 81.8)**		**0.45 (0.35, 0.58)**	**< 0.001**
**Histology**					**< 0.001**		
Other	**131**	**100 (76%)**	**9.1**	**38.7 (30.1, 47.3)**		**–**	
Adenocarcinoma	**371**	**221 (60%)**	**15.0**	**58.4 (53.3, 63.5)**		**0.57 (0.45, 0.72)**	**< 0.001**
**EC-mets**					**0.018**		
No	**273**	**166 (61%)**	**13.9**	**57.7 (51.7, 63.6)**		**–**	
Yes	**238**	**162 (68%)**	**10.5**	**47.9 (41.4, 54.5)**		**1.30 (1.04, 1.61)**	**0.018**
**WBRT**					0.11		
No	390	248 (64%)	12.2	51.0 (45.9, 56.1)		–	
Yes	131	87 (66%)	14.9	60.7 (52.3, 69.1)		0.82 (0.64, 1.05)	0.11
**Age**	**521**	**335 (64%)**	**12.7**	**53.5 (49.1, 57.8)**		**1.014 (1.003, 1.025)**	**0.012**
**KPS**	**441**	**276 (63%)**	**12.7**	**53.8 (49.1, 58.6)**		**0.96 (0.95, 0.97)**	**< 0.001**
**Lesion number**	519	333 (64%)	12.7	53.5 (49.1, 57.9)		0.99 (0.96, 1.03)	0.72
**SRS**	**509**	**327 (64%)**	**12.7**	**53.8 (49.4, 58.2)**		**0.65 (0.57, 0.75)**	**< 0.001**

Significant differences (p < 0.05) are bolded.

### ICI within 90 days

**Figure 2:**
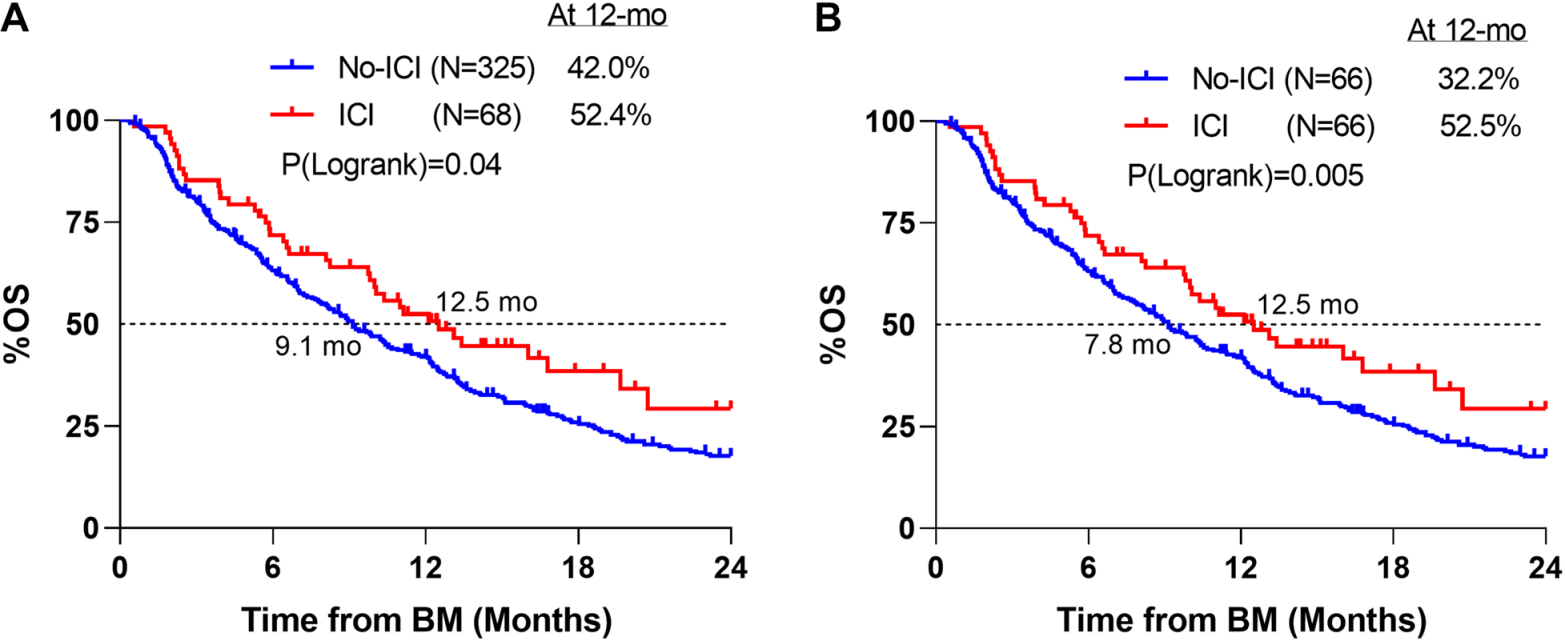
(**A**) Overall survival comparing ICI-90 to no-ICI with the exclusion of patients who received surgery for brain metastases. (**B**) After a Greedy match was performed to balance the ICI-90 and no-ICI cohort after the removal of patients with intracranial surgery, overall survival from the diagnosis of brain metastases is graphed. Log-rank test was performed.

Overall survival from the time of diagnosis of BM was compared between those treated without ICI and those treated with ICI within the first 90 days. On univariable analysis, there was a significant difference in OS between the groups with median survival of 9.1 months and 12.5 months, respectively (*p* = 0.04) (Fig. 2A). In a multivariate analysis, treatment with ICI-90 led to significantly improved OS (*p* < 0.001) (Table 3). A similar analysis was performed excluding patients with missing KPS, and ICI-90 remained a significant predictor of OS (*p* = 0.001).

**Table 3:** Multivariate analysis on the effect of ICI within 90 days.

Variable	N	Events	Cox multivariable hazard ratio (95% CI)	Cox multivariable Wald p-value
**ICI status**				
No-ICI	**325**	**247 (76%)**	**1.87 (1.30, 2.68)**	**< 0.001**
ICI-90	**68**	**39 (57%)**	**–**	
**Sex**				
Female	187	130 (70%)	1.02 (0.79, 1.32)	0.87
Male	206	156 (76%)	–	
**Age**				
< 65	**232**	**158 (68%)**	**–**	
≥ 65	**161**	**128 (80%)**	**1.302 (1.013, 1.674)**	**0.039**
**KPS**				
< 90	**178**	**138 (78%)**	**1.69 (1.28, 2.23)**	**< 0.001**
≥ 90	**141**	**93 (66%)**	**–**	
Unknown	74	55 (74%)	0.93 (0.64, 1.34)	0.69
**SRS**				
0–1	**284**	**218 (77%)**	**2.08 (1.52, 2.85)**	**< 0.001**
≥ 2	**98**	**60 (61%)**	**–**	
**Histology**				
Other	**106**	**88 (83%)**	**1.39 (1.07, 1.82)**	**0.014**
Adenocarcinoma	**268**	**184 (69%)**	**–**	
**EC-mets**				
No	**207**	**144 (70%)**	**–**	
Yes	**176**	**135 (77%)**	**1.31 (1.02, 1.68)**	**0.035**
**Lesion number**				
1	**201**	**140 (70%)**	**–**	
≥ 2	**190**	**144 (76%)**	**1.38 (1.07, 1.78)**	**0.012**

Significant differences (p < 0.05) are bolded.

Additionally, a PS matching was performed to select patients who did not receive ICIs with similar characteristics to the ICI-90 cohort. When both groups were matched to have similar age, KPS, EC-mets (exact match), number of lesions, and number of SRS treatments, there was almost a 5-month median survival advantage with ICIs within 90 days (12.5 vs 7.8 months, *p* = 0.005) (Fig. 2B).

### Impact of KRAS mutation status on the efficacy of ICI

Given the emerging data regarding presence of *KRAS* mutation and response to ICI, we first analyzed the expression of PD-L1 based on *KRAS* mutation status. Out of 800 total patients diagnosed with LCBM, 109 patients had both a known PD-L1 expression and *KRAS* status. PD-L1 was expressed in 80.4% of patients with *KRAS* mutation vs. 61.9% of *KRAS* wild-type patients (*p* = 0.04).

**Figure 3:**
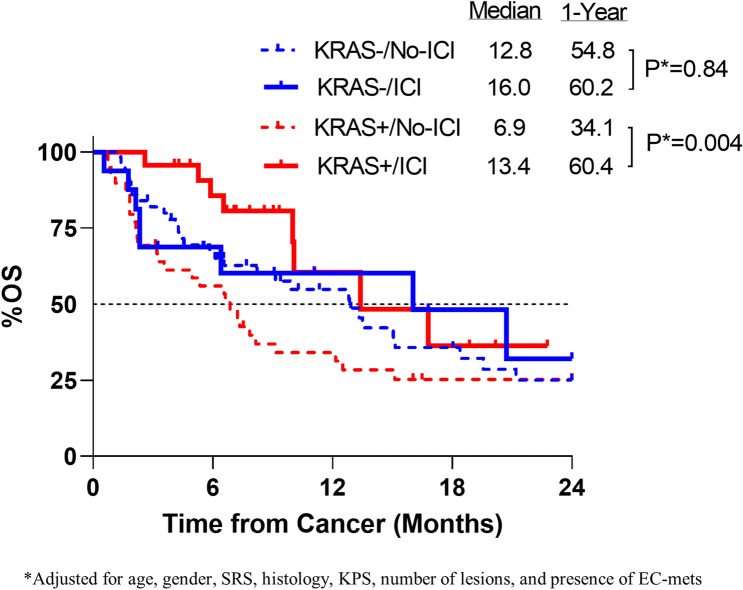
Impact of KRAS status on efficacy of ICI. Overall survival is plotted from the start of ICIs. Multivariate analysis which included age, gender, SRS, histology, KPS, number of lesions and presence of EC-mets.

We then compared OS from the time of BM between those not treated with ICIs vs. those treated with ICIs (Fig. 3). In patients without *KRAS* mutations, there was no statistical difference in OS between the ICI vs. no ICI cohorts with a one-year survival of 60.2% vs 54.8% (adjusted *p* = 0.84). However, in patients with *KRAS* mutations, patient treated with ICIs had a 1-year survival of 60.4% vs. 34.1% in the no-ICI cohort. On multivariate analysis including age, gender, SRS, histology, KPS, number of lesions, and presence of EC-mets, these results remained consistent and statistically significant (*p* = 0.004). When comparing all patients who received ICIs, there was no difference in OS from BM between patients with vs without *KRAS* mutations.

## Discussion

Patients with NSCLCBM have a poor prognosis and traditional systemic chemotherapies have provided limited clinical benefit. With advances in targeted therapies and ICI, this dogma is beginning to shift dramatically. While some clinical data is available suggesting the efficacy of ICIs in patients with stable, asymptomatic brain metastases, there is almost no prospective or retrospective data in patients with progressive or symptomatic disease. Our study retrospectively analyzed patients treated for NSCLCBM at a single tertiary care center and compared those treated with ICIs to those who were not. In agreement with the clinical trials, we observed an improvement in OS when patients were treated with ICIs within 90 days of the diagnosis of brain metastases (*p* < 0.0001)^2–4^. This data suggests that ICIs provide an OS benefit over traditional chemotherapy or local therapy alone.

Surgery has long been a mainstay of BM treatment, however, advances in SRS have decreased its utilization. While investigating the prevalence of surgical resection was not the primary goal of this study, when we compared our ICI-90 cohort to our no-ICI cohort we found a large difference in the proportion who underwent resection of a BM (29.4% vs 5.7%, *p* < 0.001) and therefore we excluded these patients from the remaining analyses. Advances in SRS technology more readily allow for the targeting of multiple intracranial lesions, deep-seated tumors, in patients who are too frail for surgery, have multiple medical co-morbidities, and is also commonly used in the salvage setting^16,17^. These improvements may account for the number of intracranial lesions not predicting OS within our cohort (hazard ratio of 0.99, *p* = 0.72). Newer prospective clinical trials are beginning to demonstrate that patients with multiple lesions can be treated with SRS and have improved intracranial control^18^. Our cohort provides additional retrospective evidence that number of lesions may not influence OS when all lesions are treated with SRS^18,19^. With that being said, other variables that have previously been reported, such as EC-mets, age and KPS all predicted OS^15,20^.

In order to identify patient populations who respond best to ICIs, we investigated the impact of *KRAS* mutation status. We found an improved OS in patients with a *KRAS* mutation who were treated with ICI within 90 days over no-ICI. This difference was not observed in the patients with wild-type *KRAS*. A similar result was seen in a meta-analysis of three clinical trials that compared the difference in response to ICIs based on *KRAS* status^21^. Additionally, another recently published study found that patients with *KRAS* mutations with advanced NSCLC had improved survival over patients with wild-type *KRAS* when treated with ICI^22^. These findings suggest that while ICIs do not directly target *KRAS* protein, they may provide significant clinical benefit to these patients. Additionally, the development of KRAS targeted therapies opens the possibility for combination therapy with ICIs^23^. *KRAS* mutation status should, therefore, be considered in the development of future clinical trials investigating ICIs and BM.

The retrospective nature of the study allows for errors in data collection from an electronic medical record not designed for research purposes. All our mutation and PD-L1 status was derived from the primary tumor and KRAS mutational subtypes were not collected. A 2015 study found that all patients with an activating KRAS mutation in the primary tumor shared this mutation in all clonally related cases^24^. Additionally, another recent study found a 75.5% concordance between PD-L1 expression in primary tumor and BM suggesting that while PD-L1 status of primary tumor has some predictive value, changes in intracranially do occur^25^. Additionally, although multivariable analysis was performed with known predictors of survival, the cohorts may have differed in other areas. We were unable to determine whether KRAS mutation status was an independent predictor of survival or if the increased PD-L1 expression in these patients accounts for the difference. The patients in the no-ICI group were not necessarily treated with any systemic chemotherapy and almost all received intracranial SRS. Finally, while our study investigated OS after BM, we did not specifically investigate intracranial or extracranial response, allowing the possibility that any difference in OS may be due to control (or lack thereof) of extracranial disease.

This study reports a significant survival benefit of ICIs over chemotherapy or local control alone in patients with NSCLCBM. This retrospective analysis of patients treated for NSCLCBM has also emphasized the shift away from surgery for management of BM in the age of ICIs. Finally, our data suggests the greatest benefit of ICIs are seen in patients with *KRAS* mutations in their tumors, which should be considered in future trials.
