# Author Correction: Impact of KRAS mutation status on the efficacy of immunotherapy in lung cancer brain metastases

**DOI:** 10.1038/s41598-022-05489-0

**Published:** 2022-01-17

**Authors:** Adam Lauko, Rupesh Kotecha, Addison Barnett, Hong Li, Vineeth Tatineni, Assad Ali, Pradnya Patil, Alireza M. Mohammadi, Samuel T. Chao, Erin S. Murphy, Lilyana Angelov, John H. Suh, Gene H. Barnett, Nathan A. Pennell, Manmeet S. Ahluwalia

**Affiliations:** 1 Case Western Reserve University School of Medicine MSTP, Cleveland, OH USA; 2 Department of Radiation Oncology, Miami Cancer Institute, Baptist Health South Florida, Miami, FL USA; 3 Herbert Wertheim College of Medicine, Florida International University, Miami, FL USA; 4 Rosa Ella Burkhart Brain Tumor and Neuro-Oncology Center, Taussig Cancer Institute, Cleveland, OH USA; 5 Department of Quantitative Health Sciences, Lerner Research Institute, Cleveland Clinic, Cleveland, OH USA; 6 Department of Internal Medicine, Summa Health, Akron City Hospital, Akron, OH USA; 7 Department of Medical Oncology, Taussig Cancer Institute, Cleveland Clinic, Cleveland, OH USA; 8 Department of Neurological Surgery, Neurological Institute, Cleveland Clinic, Cleveland, OH USA; 9 Department of Radiation Oncology, Taussig Cancer Institute, Cleveland Clinic, Cleveland, OH USA; 10 Department of Medical Oncology, Miami Cancer Institute, Baptist Health South Florida, 8900 North Kendall Drive, Miami, FL 33176 USA

Correction to: *Scientific Reports* https://doi.org/10.1038/s41598-021-97566-z, published online 13 September 2021

The original version of this Article contained an error in the Funding section.

“This research received no specific grant from any funding agency in the public, commercial, or not-for-profit sectors.”

now reads:

“This work was partially funded by the Ruth L. Kirschstein National Research Service Award (NRSA) Individual Fellowship F30CA250254 (AL).”

The original Article has been corrected.

